# Plasticizer-Enabled Solvent-Free Curing of Self-Healing Binder System for Energetic Materials

**DOI:** 10.3390/polym17192635

**Published:** 2025-09-29

**Authors:** Minghao Zhang, Xudong Hou, Qifa Yao, Hanyu Chen, Zuting Wei, Yue Zhao, Zhishuai Geng, Fanzhi Yang, Min Xia, Yunjun Luo

**Affiliations:** 1 School of Materials Science and Engineering, Beijing Institute of Technology, Beijing 100081, China; 3120245748@bit.edu.cn (M.Z.);; 2 Advanced Research Institute of Multidisciplinary Science, Beijing Institute of Technology, Beijing 100081, China; 3 Key Laboratory of High Energy Density Materials, Ministry of Education, Beijing Institute of Technology, Beijing 100081, China

**Keywords:** self-healing, plasticizer, hydroxyl-terminated polybutadiene, solid propellant, solvent-free curing

## Abstract

Solvent processing hampers the reliability and energy density of self-healing binders for energetic materials. We report a solvent-free curing route for a Diels–Alder self-healing furanyl-terminated polybutadiene enabled by a functional external plasticizer, dibutyl phthalate (DBP), which acts not only to lower the viscosity of the binder but to disperse the high-melting bismaleimide, thereby driving crosslinked network formation. The 50 wt% DBP-plasticized film healed a pre-cut crack in 5 min at 120 °C and recovered nearly full mechanical properties after 24 h at 60 °C. Based on this binder system, a self-healing solid propellant with 80 wt% solid content was solvent-free cast into a dense and void-free grain that healed surface cracks within 5 min at 120 °C. This solvent-free approach overcomes the limitations of solvent-based processing and offers a viable fabrication route for self-healing energetic materials.

## 1. Introduction

Energetic materials are substances that contain stored chemical energy released rapidly upon initiation—typically explosives, propellants, and pyrotechnic compositions—and within such formulations, polymers primarily serve as binders that impart cohesion, mechanical integrity, processability, and sensitivity reduction. Nevertheless, polymers are susceptible to microcracks and structural defects that accumulate during service, often leading to a noticeable decline in their functional lifetime [1]. Although thermoplastic welding, adhesive repair, or low-cost replacement are viable for many commodity polymers, they are frequently unsuitable for energetic systems, where the polymer is usually a crosslinked, particle-rich binder cast in sealed hardware and subject to strict safety requirements. Consequently, in situ self-healing that restores integrity without disassembly is particularly appealing for energetic materials.

Drawing inspiration from the intrinsic self-repair capabilities of biological systems [2,3], the design of self-healing polymer materials (SPMs) aims to achieve restoration of structural integrity upon damage, thus extending service life and improving sustainability [4,5,6,7,8]. SPMs are broadly classified into extrinsic and intrinsic healing mechanisms [9]. Extrinsic healing mechanisms typically rely on the release of encapsulated healing agents upon crack propagation [10,11,12], whereas intrinsic counterparts leverage dynamic reversible interactions—such as Diels–Alder reactions (DA) [13,14], disulfide exchange reactions [15,16,17], transesterification [18,19], hydrogen bonding [20,21,22], and metal–ligand coordination [23,24]—to endow polymer chains with repeatable healing functionality and high universality, making them the focus of increasing research interest [25,26,27,28]. As energetic materials are particle-filled polymer-based composites and their fabrication processes typically involve high-shear mixing, introducing intrinsic self-healing is often the more suitable approach.

Despite recent advances, the implementation of intrinsic SPMs remains largely dependent on solvent-assisted curing methods, primarily because of the high melting points and low miscibility of certain reactive components. Reagents such as 2,6-pyridinedimethanol (melting point > 112 °C) [29] and piperazine (melting point > 109 °C) [30] exhibit poor dispersibility in the absence of solvents, which often results in incomplete curing and suboptimal polymer network formation. Moreover, the high viscosity of prepolymer matrices complicates solvent-free processing, limiting the curing uniformity and reaction efficiency.

However, in energetic materials, uncontrolled evaporation during solvent-based processing often yields inhomogeneous microstructures, bubble formation, and batch-to-batch variability; moreover, the introduction of large amounts of solvent reduces the solid content and thereby directly diminishes the formulation’s energy density, so most reported systems are limited to low solid loading self-healing energetic materials [31,32]. In our previous work, we developed a DA-based intrinsic self-healing furanyl-terminated polybutadiene (FTPB) [33]. Because the curing agent bismaleimide (BMI) has a high melting point, the system depended on solvent processing [34,35,36], which posed serious challenges for preparing high solid loading self-healing energetic materials.

To address these limitations, we proposed a solvent-free processing strategy by introducing an appropriate external plasticizer as a functional substitute for traditional solvents. External plasticizers are low-volatility additives used to lower prepolymer viscosity and enhance processability, while remaining chemically inert with respect to reactive components [37,38], and they are widely employed as functional additives in energetic materials. Herein, we systematically investigated the effect of several typical plasticizers on the FTPB system, evaluating their ability to enhance component dispersion and promote curing behavior. Using the optimized plasticizer, we intend to develop a solvent-free curing system with excellent processability and self-healing performance and to produce high solid loading solid propellants through a simple solvent-free mixing-casting process, thereby advancing a design strategy for constructing solvent-free curing intrinsic self-healing materials and facilitating their scalable application in energetic materials.

## 2. Experimental

### 2.1. Materials

Hydroxyl-terminated polybutadiene (HTPB, type IV, M_n_ = 3210 g/mol, vacuum-dried at 60 °C for 48 h), isophorone diisocyanate (IPDI, 99%), and dibutyltin dilaurate (DBTDL, 95%) were purchased from Macklin Biochemical Co., Ltd. (Shanghai, China). Bismaleimide (BMI, 98%), dibutyl phthalate (DBP, 99%), bis(2-ethylhexyl) adipate (DOA, 99%), and bis(2-ethylhexyl) sebacate (DOS, 97%) were obtained from Meryer Co., Ltd. (Shanghai, China). N-n-butyl-N-(2-nitroxyethyl) nitramine (Bu-NENA, 99%) was supplied by Liming Chemical Research Institute (Luoyang, China). Furfurylamine (FA, 99%) and tetrahydrofuran (THF, 99.9%) were purchased from J&K Scientific Ltd. (Beijing, China). Epoxidized soybean oil (ESBO, CP) was purchased from Aladdin (Shanghai, China). Aluminum powder (D_50_ = 24.86 µm, dried at 60 °C for 7 d) was purchased from Hunan Xiangtou Lightweight Material Technology Co., Ltd. (Changsha, China). Ammonium perchlorate (AP, 99.8%, dried at 60 °C for 7 d; Class I and Class III with mean particle sizes of 335 µm and 123 µm, respectively) was purchased from Dalian Gaojia Chemical Co., Ltd. (Dalian, China).

### 2.2. Synthesis of FTPB Binder

**Scheme 1:**
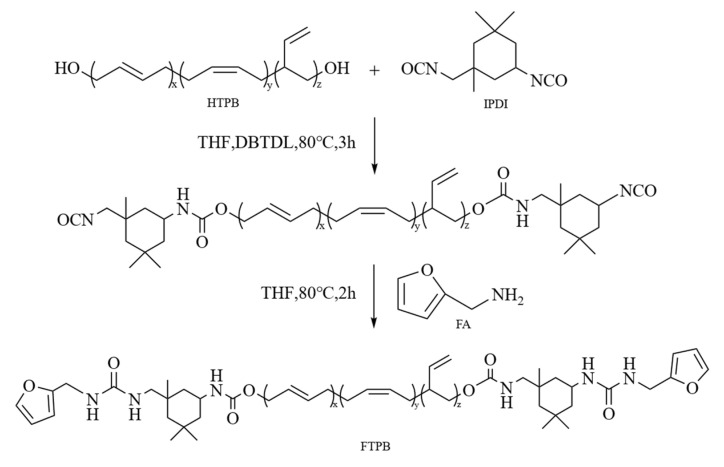
Synthetic pathway of FTPB.

The synthetic pathway [33] of FTPB is illustrated in Scheme 1. HTPB was first dissolved in THF. IPDI was then added at a molar ratio of -OH to -NCO of 1:2, and the mixture reacted at 80 °C for 3 h in the presence of DBTDL as a catalyst, yielding isocyanate-terminated polybutadiene. After cooling the solution to 20 °C in an ice-water bath, FA was added dropwise in an equimolar amount relative to the residual -NCO groups. The mixture was stirred magnetically at 20 °C for 30 min, followed by continued reaction at 80 °C for an additional 2 h. FTPB was obtained after purification by holding the crude product under vacuum at 60 °C for 48 h.

### 2.3. Solvent-Free Curing of FTPB

**Scheme 2:**
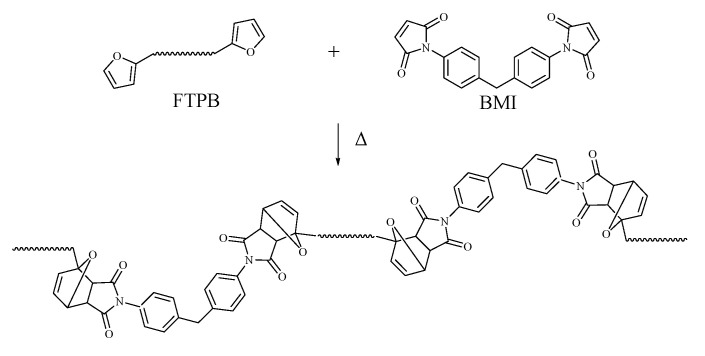
Curing pathway of FTPB.

The curing pathway of FTPB with BMI is shown in Scheme 2. FTPB was blended with a plasticizer at a predetermined mass ratio and mechanically stirred for 10 min, then held at 60 °C for 30 min without agitation. Unless otherwise specified, the plasticizer weight fractions are defined relative to the combined mass of the plasticizer and FTPB. To ensure sufficient diffusion of plasticizer molecules into the polymer network, the stir–hold steps were repeated two times. Subsequently, BMI was added at a molar amount equal to half that of the furan groups in the system. The mixture was stirred for 10 min and cured at 70 °C for 48 h to yield a self-healing film. It is noteworthy that the plasticizer employed in this step must exhibit good compatibility with FTPB to ensure long-term phase stability of the blend without stratification.

### 2.4. Preparation of Self-Healing Solid Propellant (SHSP)

To the mixture of FTPB, plasticizer, and BMI from Section 2.3, aluminum powder (17 wt%) and AP (33 wt% Class I and 30 wt% Class III) were added in sequence and mixed thoroughly until homogeneous. This propellant slurry was degassed under vacuum at 40 °C for 3 h, then held at 100 °C for 30 min, and finally cured at 70 °C for 48 h to obtain SHSP.

### 2.5. Characterization Methods

Viscosity measurements were conducted using an R/S-SST Plus rheometer (Brookfield, Middleboro, MA, USA) equipped with a 50 mm parallel-plate rotor at a gap of 0.5 mm. Viscosity at a shear rate of 12.5 s^−1^, within the processing-relevant range for mixing during propellant manufacture, was taken from the steady-state region of the flow curve to minimize transients and wall slip. Samples were pre-sheared for 20 s, and the apparent viscosities were measured at a shear rate of 12.5 s^−1^ under temperatures of 25 °C and 60 °C. The crosslink density was determined using low-field nuclear magnetic resonance spectroscopy (LF-NMR) VTMR20-010V-T (Niumag Corporation, Suzhou, China) at a test temperature of 90 °C. Each measurement was repeated three times per sample, and the average value was reported. The self-healing behavior of films and propellants was observed using a GP-200MRT high-definition metallographic microscope (Gaopin Precision Instrument Co., Ltd., Kunshan, China). Mechanical properties were measured on a Shimadzu AGS-J universal testing machine (Shimadzu Co., Ltd., Kyoto, Japan). Dumbbell-shaped specimens were prepared with a gauge section of ~12 mm × 2 mm × 2 mm, and the crosshead speed was 100 mm/min. Differential scanning calorimetry (DSC) was performed on DSC3+ from METTLER TOLEDO (Zurich, Switzerland) under an N_2_ atmosphere. The solubility of BMI was determined at each set temperature by stepwise addition of 0.05 g BMI to 50.0 g plasticizer until the onset of Tyndall scattering.

## 3. Results and Discussion

### 3.1. Selection of Plasticizer

**Figure 1:**
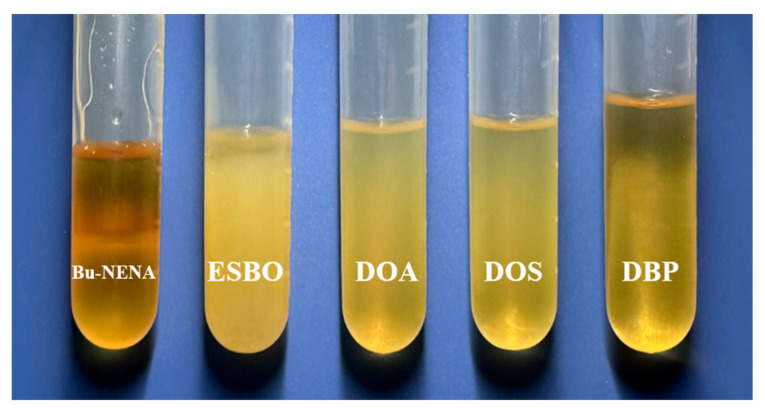
FTPB mixed with plasticizers after standing at room temperature for 10 min.

Five plasticizers commonly used in polymer and energetic materials were selected for compatibility evaluation with FTPB. Each plasticizer was thoroughly mixed with FTPB at a mass ratio of 1:1, and the mixtures were allowed to stand at room temperature for 10 min. Representative photographs of the resulting mixtures are shown in Figure 1. Apparent phase separation was observed in the mixture containing Bu-NENA, indicating poor miscibility with FTPB. The mixture with ESBO rapidly solidified at room temperature, suggesting unfavorable interactions. In contrast, FTPB blended with DOS, DOA, or DBP remained homogeneous at room temperature, indicating good compatibility.

**Figure 2:**
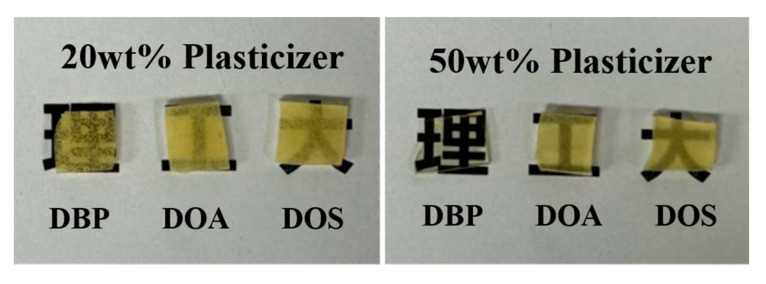
FTPB films plasticized with DBP, DOA, and DOS.

FTPB films plasticized with DOS, DOA, or DBP were prepared, with their macroscopic appearances shown in Figure 2. DOS- and DOA-plasticized films exhibited low optical transparency, attributable to incomplete reaction of BMI, which remained in the matrix as particulate matter. Such behavior is expected, given that plasticizers generally possess inferior solubilizing power for solid reagents compared with traditional solvents. Surprisingly, a different trend was observed for the DBP-plasticized system. The film containing 20 wt% DBP exhibited moderate transparency, while the 50 wt% DBP film appeared nearly transparent. This suggests that, under DBP plasticization, the curing reaction of the self-healing system proceeded to a significantly higher extent than in the DOS- and DOA-containing systems. In this case, DBP functioned not only as a plasticizer but also as an effective medium facilitating the dispersion of BMI, behaving similarly to a conventional solvent. Enhanced curing behavior can be attributed to two effects: (i) matrix plasticization, which lowers viscosity and increases chain mobility of the prepolymer [39], and (ii) improved wetting and dispersion of solid BMI, which enlarges the reactive interface.

**Figure 3:**
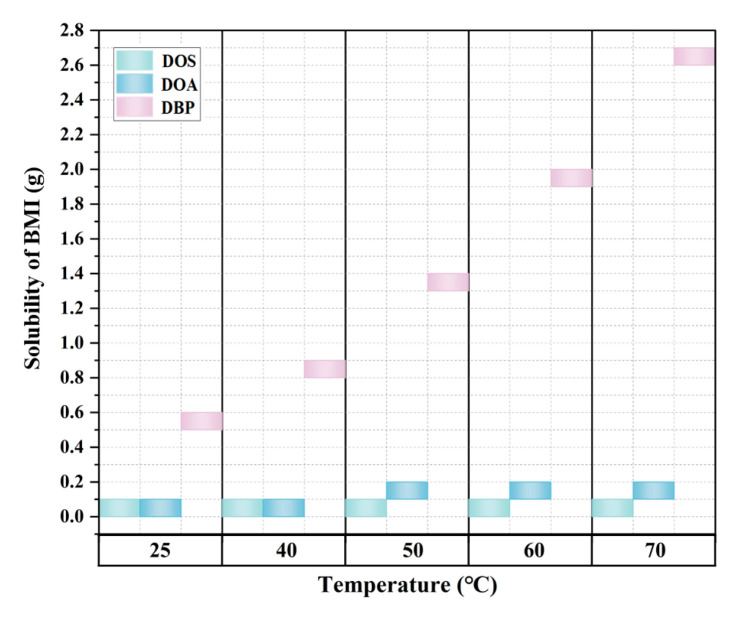
Solubility of BMI in DBP, DOA, and DOS at different temperatures.

A higher degree of dispersion of BMI within the system allows more BMI molecules to interact with reactive sites on the polymer chains, thereby promoting a more complete curing process. Figure 3 presents the solubility of BMI in DBP, DOA, and DOS at different temperatures. These data reveal that DBP exhibits a much higher capacity for dissolving BMI compared with DOA and DOS. The DBP molecule contains a rigid, planar phenyl core with two ester-bearing side chains [40]; together with the imide ring and aromatic units of BMI [41], it can engage in π-π stacking, leading to pronounced intermolecular association, whereas DOS and DOA lack this capability. This enhanced dispersion is considered a key factor contributing to the near-transparency of the films plasticized with DBP. At the early stage of curing, due to the limited amount of plasticizer present, most of the BMI remains undissolved, resulting in a turbid system. As the curing reaction proceeds, BMI molecules that are either dissolved in the plasticizer or located at the outer layers of BMI particles begin to react with the polymer chains, gradually forming covalent bonds and being consumed. This process leads to the progressive deagglomeration and reaction of the remaining BMI particles. When the plasticizer has sufficient dispersion capability, a substantial fraction of BMI can ultimately participate in network formation with the prepolymer, resulting in a significant improvement in film transparency after curing.

**Figure 4:**
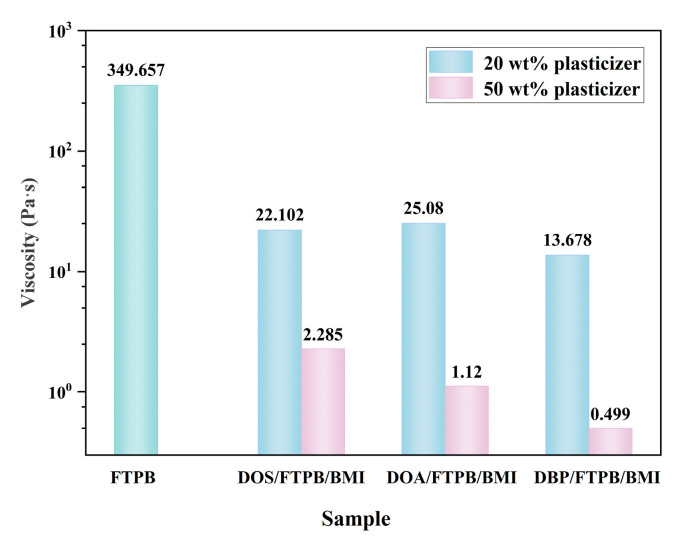
Viscosity of FTPB systems at 60 °C and a shear rate of 12.5 s^−1^.

The plasticizing effects of DBP, DOA, and DOS on the FTPB system were evaluated via viscosity measurements. Figure 4 shows the viscosities of the blends measured at 60 °C and a shear rate of 12.5 s^−1^. All three plasticizers markedly lowered the viscosity, with DBP being the most effective. In the 50 wt% DBP blend, the viscosity fell to less than 1/700 of the pure FTPB value. Such a substantial reduction in viscosity markedly improves the processability of the solvent-free system [42]. By intercalating between polymer chains, plasticizer molecules reduce intermolecular interactions and increase segmental mobility, thereby facilitating diffusion and reaction with BMI and increasing the extent of curing.

**Scheme 3:**
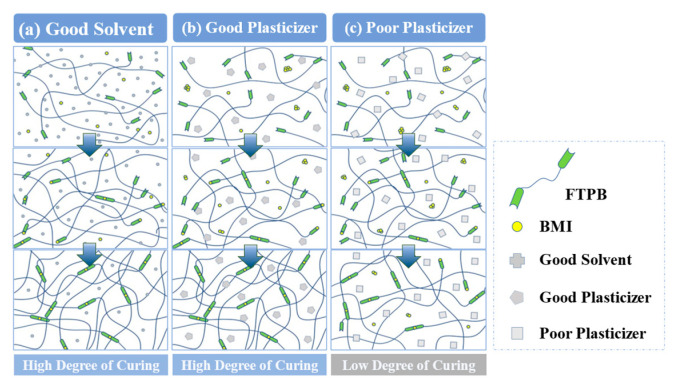
Curing behavior of FTPB systems under different environments: (**a**) good solvent, (**b**) good plasticizer (e.g., DBP), and (**c**) poor plasticizer (e.g., DOS or DOA).

Scheme 3 contrasts FTPB systems curing in three media—a good solvent, a good plasticizer, and a poor plasticizer. In a good solvent, BMI dissolves uniformly to give clear films; plasticizer systems are initially turbid, but a good plasticizer promotes BMI dispersion and reaction, thereby improving the transparency of the cured films. Notably, this strategy extends beyond FTPB-based self-healing systems and can inform the design of other solvent-free curing systems with similar constraints, including those outside the self-healing domain.

### 3.2. Properties of DBP-Plasticized FTPB Films

**Figure 5:**
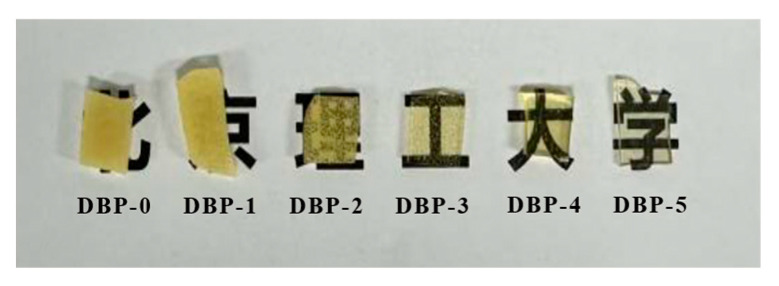
FTPB films with varying DBP content.

**Figure 6:**
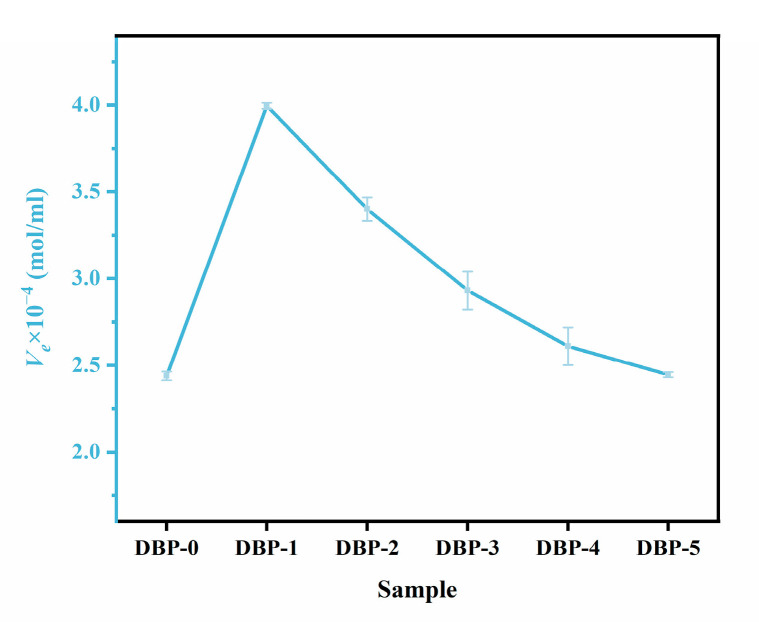
Crosslink density (*V_e_*) of DBP-plasticized FTPB films.

Following the identification of DBP as an effective plasticizer for the self-healing FTPB system, a series of films was prepared with DBP mass fractions of 0 wt%, 10 wt%, 20 wt%, 30 wt%, 40 wt%, and 50 wt%, respectively, designated as DBP-0 through DBP-5. The macroscopic appearance of the films is shown in Figure 5. As the DBP content increased, the films became increasingly transparent. The extent of curing can be qualitatively assessed by characterizing the film’s crosslinked network. The main parameter that characterizes the structure of a polymer crosslinking network is the crosslinking density, which can be determined by LF-NMR [43]. Figure 6 shows the crosslink density of each film. When the DBP content increased from 0 wt% to 10 wt%, the crosslink density increased substantially, indicating a significant enhancement in the curing efficiency attributable to the presence of DBP. While higher DBP contents continued to promote curing, the concomitant reduction in the absolute amounts of FTPB and BMI gradually lowered the overall crosslink density. Nevertheless, the DBP-5 film, although containing only half as much FTPB and BMI as DBP-0, still exhibited a higher crosslink density than DBP-0, further confirming the beneficial role of DBP in facilitating network formation during solvent-free curing. For this reason, the number of visible BMI particles in the film decreases with increasing DBP content.

**Scheme 4:**
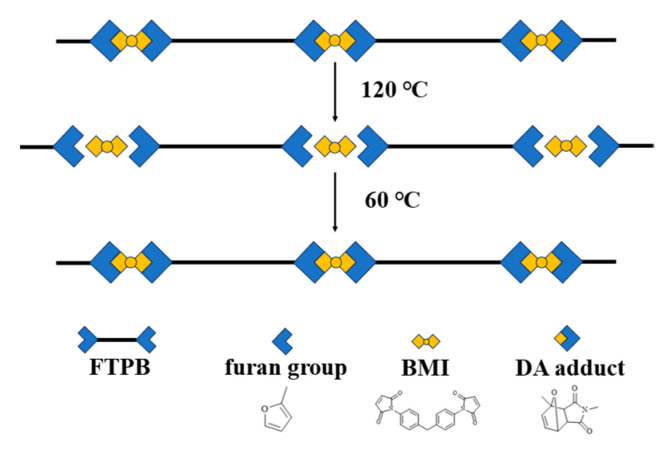
Self-healing mechanism of FTPB films via reversible Diels–Alder reaction.

**Figure 7:**
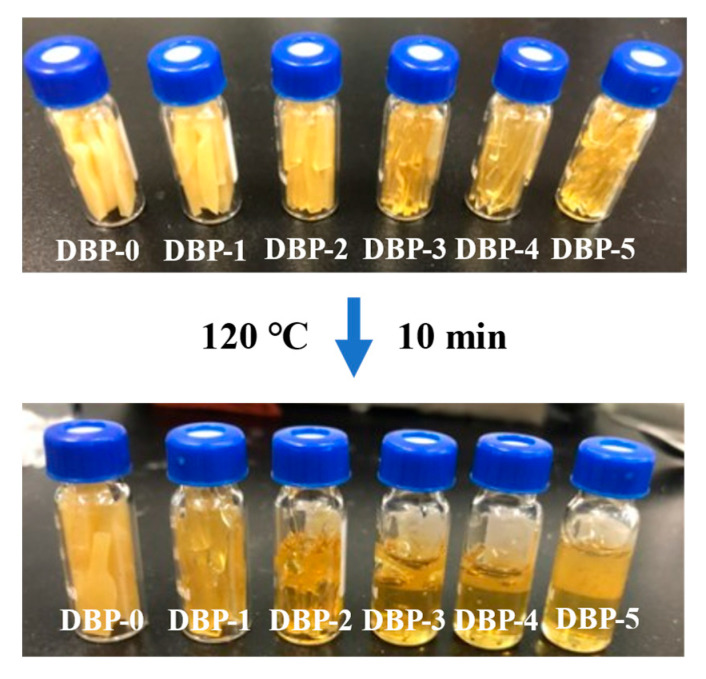
Recyclability of DBP-plasticized FTPB films after heating at 120 °C for 10 min.

Scheme 4 illustrates the self-healing mechanism of FTPB films. Upon heating to 120 °C, the retro-Diels–Alder (rDA) reaction is triggered, cleaving the covalent network and regenerating FTPB and BMI [44]. These molecular fragments diffuse into the crack through thermal motion and reoccupy the damaged regions. Subsequently, the specimen was held at 60 °C for 24 h to drive the forward Diels–Alder reaction, reforming covalent bonds between FTPB and BMI and thereby restoring mechanical integrity [45]. The efficiency of molecular diffusion following the rDA reaction strongly influences the self-healing rate and the recyclability of the material. To investigate this, films DBP-0 through DBP-5 were cut into strips and placed in a container, as shown in Figure 7. After heating at 120 °C for 10 min, significant morphological changes were observed in DBP-2 to DBP-5, indicating enhanced recyclability. The extent of this morphological transformation positively correlated with DBP content, consistent with the role of plasticizers in enhancing chain mobility. The plasticizer, distributed between polymer chains, facilitates chain migration and diffusion of reactive species after bond cleavage, enabling rapid crack closure and reshaping at the macroscopic level. For self-healing films cured in solvent-rich environments, rapid solvent evaporation during curing increases matrix viscosity, often necessitating external pressure (>5 MPa) and prolonged times (>30 min) to enable recycling [46]. Alternatively, the polymer must be redissolved in the solvent at an elevated temperature and then re-cured by solvent re-evaporation [47]. In contrast, the solvent-free curing strategy employed in this study retains plasticizers within the network post-curing, allowing the matrix to maintain low viscosity after rDA reaction. This significantly enhances molecular mobility and recyclability, enabling fast reprocessing without the need for external pressure.

**Figure 8:**
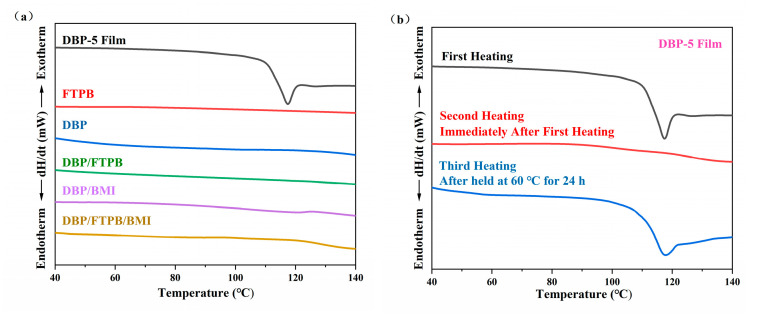
Heat-flow curves for (**a**) DBP-5 film, raw materials, and their mixture (DBP-5 ratio) and (**b**) DBP-5 film upon repeated heating.

Self-healing performance was evaluated for the DBP-5 film, which exhibited the best processability and recyclability. The thermal behavior upon heating was examined for the corresponding raw materials and their mixture formulated at the DBP-5 ratio. As shown in Figure 8a, the DBP-5 film exhibits an endothermic peak [48] associated with the rDA reaction at ~105–122 °C, whereas neither the individual raw materials nor their physical mixture displays this feature. When the same DBP-5 specimen was rescanned shortly after the first run, the rDA endothermic peak disappeared (Figure 8b), because the DA reaction had insufficient time to reform the adduct. After holding the twice-scanned sample at 60 °C for 24 h, a third heating run again revealed the rDA endothermic peak, confirming reformation of the DA adduct structure. In addition, the lack of an endothermic peak during the second heating supports the conclusion that the peak is not the result of film melting.

**Figure 9:**
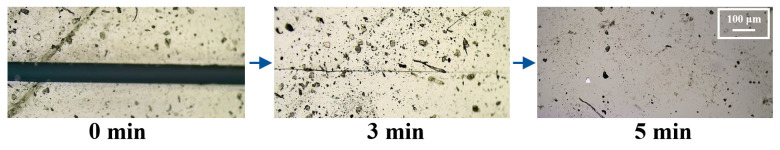
Healing process of the DBP-5 film.

**Figure 10:**
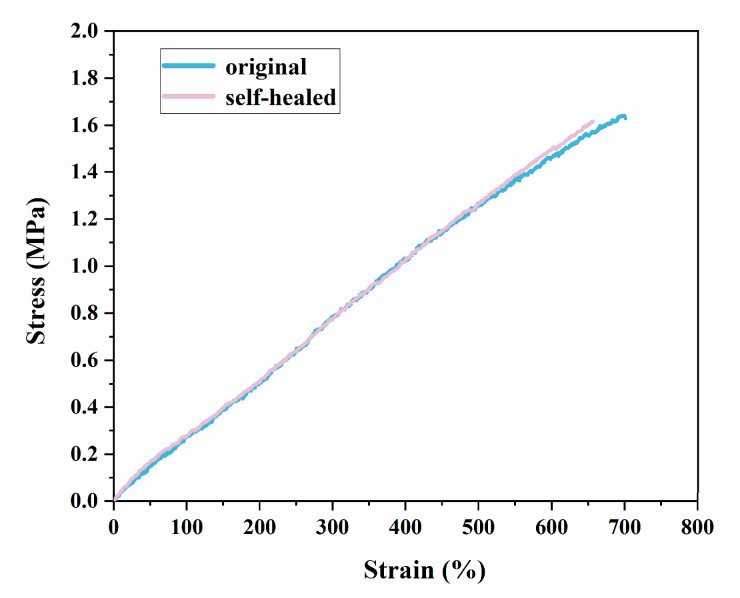
Stress–strain curves of DBP-5 before and after healing.

The DBP-5 films were nearly severed using a surgical scalpel, leaving only a minimal physical connection to ensure alignment. The self-healing behavior was observed under a polarized optical microscope, as shown in Figure 9. At 120 °C, the DBP-5 film achieved rapid crack healing within 5 min. To minimize deformation during thermal healing, either tightly control the healing time or fix the film in a mold to retain its shape. Figure 10 shows the stress–strain curves of DBP-5 before and after healing; the two curves nearly overlap, indicating excellent self-healing performance. The healed specimen shows a slightly higher tensile strength and a slightly lower elongation at break than before healing, which is attributed to high-temperature-induced dissolution of trace unreacted BMI into the liquid phase, ultimately increasing the film’s crosslink density.

### 3.3. Performance of SHSP

**Figure 11:**
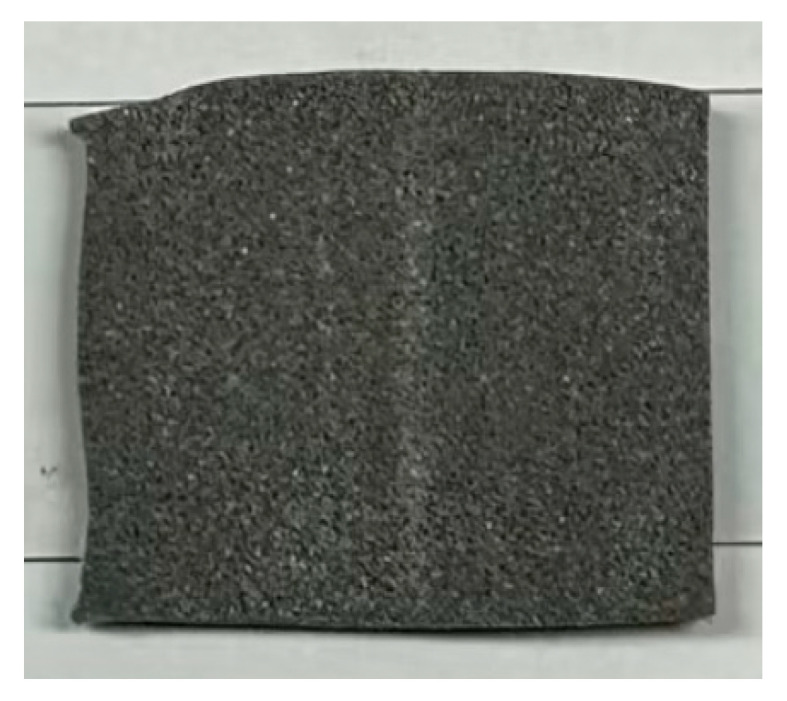
Longitudinal section of SHSP.

**Figure 12:**
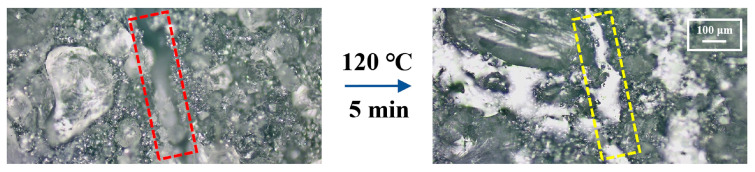
Healing process of SHSP.

Using the DBP-5 binder formulation, an SHSP with 80 wt% solid loading was prepared by solvent-free casting. The longitudinal section of the SHSP (Figure 11) is uniform and dense, with no visible voids or bubbles. Consistent with DBP-5, the SHSP also exhibits rapid crack healing; its appearance before and after healing is shown in Figure 12. Despite the high solid loading of the energetic composite, the binder provides sufficient driving force for healing, enabling a pre-formed crack to heal within 5 min at 120 °C, owing to the excellent processability and self-healing capability of the DBP-5 formulation.

**Figure 13:**
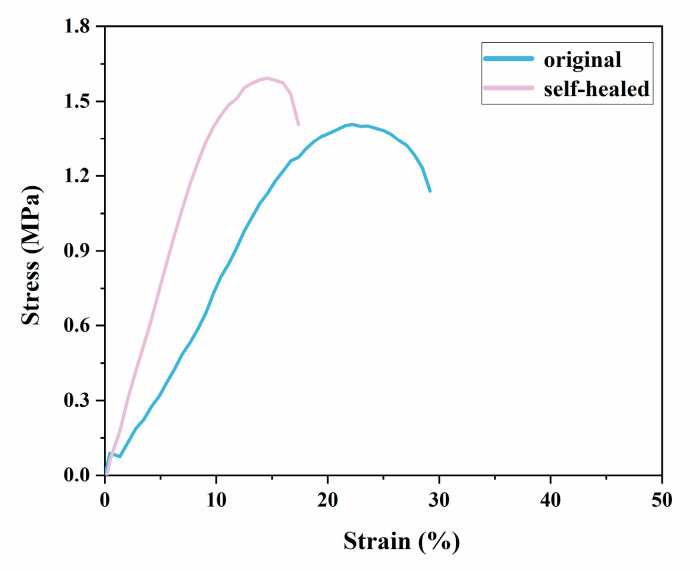
Stress–strain curves of SHSP before and after healing.

Figure 13 shows the stress–strain curves of the SHSP before and after healing. After healing a pre-formed crack at 120 °C, the SHSP was held at 60 °C for 24 h and regained mechanical strength. Compared with the original specimen, the healed SHSP exhibits a lower elongation at break and a higher tensile strength; this trend is more pronounced than in the DBP-5 film. This suggests that solid fillers partially hinder the transfer of BMI into the liquid phase, leaving more unreacted BMI particles. During the high-temperature rDA step, these particles enter the liquid phase, increasing the crosslink density of the SHSP and producing the observed changes in properties. The crosslink densities before and after healing were 4.196 × 10^−4^ and 4.584 × 10^−4^ mol/mL, respectively, supporting this mechanism.

## 4. Conclusions

This work demonstrates that a judiciously chosen external plasticizer can function as a dispersion medium to enable solvent-free curing of the DA-based self-healing FTPB system. DBP was identified as the optimal choice among the candidates screened, because it simultaneously (i) shows good compatibility with FTPB at room temperature and (ii) uniform dispersion of the high-melting BMI curing agent by π-π stacking, yielding higher curing extent than DOA and DOS under identical conditions. Leveraging the reversible Diels–Alder reaction, the optimized plasticized binder system (DBP-5 formulation) exhibited rapid crack healing at 120 °C and achieved near-complete recovery of mechanical properties after 24 h at 60 °C, demonstrating robust self-healing performance and recyclability. Extending this strategy to energetic materials, we successfully solvent-free cast SHSP with an 80 wt% solid loading, yielding a dense and void-free grain and validating the feasibility of this approach for preparing self-healing energetic materials. Future work will systematically evaluate the combustion and safety performance of SHSP with pre-introduced damage before and after healing.

## Data Availability

The original contributions presented in this study are included in the article. Further inquiries can be directed to the corresponding author.
